# Shape - but Not Size - Codivergence between Male and Female Copulatory Structures in *Onthophagus* Beetles

**DOI:** 10.1371/journal.pone.0028893

**Published:** 2011-12-14

**Authors:** Anna L. M. Macagno, Astrid Pizzo, Harald F. Parzer, Claudia Palestrini, Antonio Rolando, Armin P. Moczek

**Affiliations:** 1 Dipartimento di Biologia Animale e dell'Uomo, Università degli Studi di Torino, Torino, Italy; 2 Department of Biology, Indiana University, Bloomington, Indiana, United States of America; 3 Centro Nazionale per lo Studio e la Conservazione della Biodiversità Forestale “Bosco Fontana” di Verona, Corpo Forestale dello Stato, Mantova and Verona, Italy; 4 Dipartimento di Biologia e Biotecnologie “Charles Darwin”, Sapienza Università di Roma, Roma, Italy; Michigan State University, United States of America

## Abstract

Genitalia are among the fastest evolving morphological traits in arthropods. Among the many hypotheses aimed at explaining this observation, some explicitly or implicitly predict concomitant male and female changes of genital traits that interact during copulation (i.e., lock and key, sexual conflict, cryptic female choice and pleiotropy). Testing these hypotheses requires insights into whether male and female copulatory structures that physically interact during mating also affect each other's evolution and patterns of diversification. Here we compare and contrast size and shape evolution of male and female structures that are known to interact tightly during copulation using two model systems: (a) the sister species *O. taurus* (1 native, 3 recently established populations) and *O. illyricus*, and (b) the species-complex *O. fracticornis-similis-opacicollis*. Partial Least Squares analyses indicated very little to no correlation between size and shape of copulatory structures, both in males and females. Accordingly, comparing shape and size diversification patterns of genitalia within each sex showed that the two components diversify readily - though largely independently of each other - within and between species. Similarly, comparing patterns of divergence across sexes showed that relative sizes of male and female copulatory organs diversify largely independent of each other. However, performing this analysis for genital shape revealed a signature of parallel divergence. Our results therefore suggest that male and female copulatory structures that are linked mechanically during copulation may diverge in concert with respect to their shapes. Furthermore, our results suggest that genital divergence in general, and co-divergence of male and female genital shape in particular, can evolve over an extraordinarily short time frame. Results are discussed in the framework of the hypotheses that assume or predict concomitant evolutionary changes in male and female copulatory organs.

## Introduction

Arthropod genitalia have generally complex form and evolve more rapidly than external traits, to the extent that many species can be recognised reliably only on the basis of genital morphology. Morphological modifications of copulatory organs are therefore thought to play a major role in reproductive isolation and speciation [1]. Efforts to better understand the mechanisms underlying the unusual pattern of morphological complexity and rapid divergence has generated considerable debate, and several important hypotheses have been put forward, focusing on both natural (lock and key, pleiotropy) and sexual (sexy sons, good genes, sperm competition or sexual conflict) selection (reviewed in [2]). In general, most authors agree in considering sexual selection as the driving force underlying the striking morphological variability of genitalia [1], [3].

Another peculiarity of copulatory structures is that they generally show negative static allometries. This is usually interpreted as evidence of a certain constancy of their size with respect to body size, and explained on the basis of the one-size-fits all hypothesis [4] or a more general version thereof, that includes both mechanical fit and stimulation [5].

Most hypotheses on genitalic evolution have been developed and tested on highly male-biased datasets. Data on females are far less extensive; in general, they too suggest a similar trend of low allometric slopes, that may also be explained at first glance by mechanical fit and stimulatory one-size-fits-all arguments [5]. Nevertheless, female genitalia also seem to be less variable than male genitalia when compared between species [1], which may contradict hypotheses of genital evolution that imply concomitant male-female changes. Despite the paucity of data on females, several prominent hypotheses explicitly or implicitly assume concomitant male and female changes of genital traits that interact during copulation [2], including (1) lock and key (which implies a female's ability to *exclude* mechanically intromittent organs, avoiding sperm transfer to prevent interspecific mating), (2) mechanical conflict of interest over mating (which posits that whenever there is polygamy and reproduction is costly, genitalia evolution may be shaped by an evolutionary arms race between sexes for control over reproduction), (3) pleiotropy (which suggests that male and female genitalia share some of their genetic basis with other structures) and (4) sexual selection by cryptic female choice (which proposes that the morphology of female copulatory traits affects how they perceive stimuli during copula and that females select sperm of males that provide the right tactile stimuli during mating due to their genital morphology). Here we investigate patterns of male and female genitalic evolution in the horned beetle genus *Onthophagus*.

**Figure 1:**
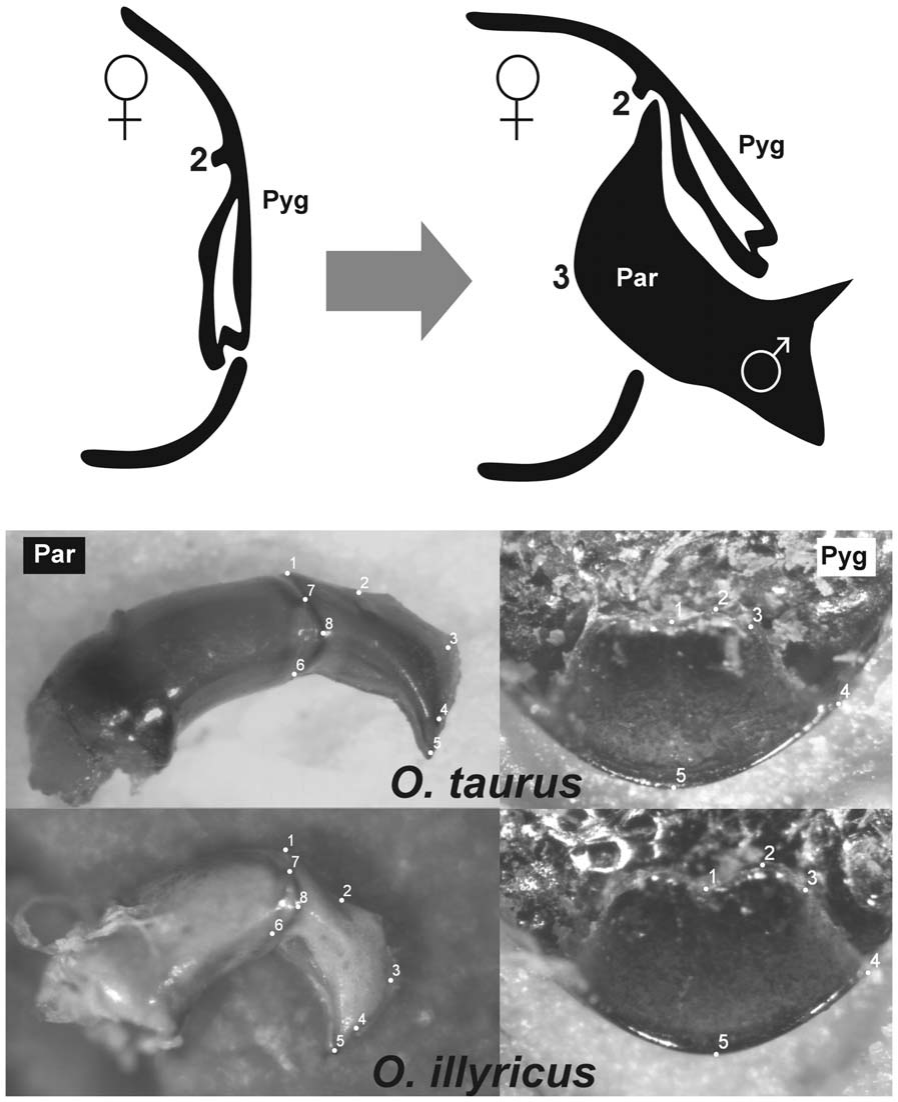
Top: schematic representation of the interaction of male paramere (Par) and female pygidial flap (Pyg) during copulation in *O. taurus* (cross section of the distal portion of female abdomen, redrawn after [**12**]). For easier visualisation, the location of landmark 2 of pygidial flap and 3 of paramere are shown. Bottom: Landmark configurations used to describe the shape of parameres and pygidial flaps (Pyg) in *O. taurus* and *O. illyricus*.

*Onthophagus* is a highly speciose and morphologically diverse genus. Recent studies on the evolution of genitalia in this genus have focused on the role of sperm competition and, in general, of sexual selection [6]–[8], as well as on tradeoffs in the development of male copulatory organs and secondary sexual traits [9], [10]. Several studies have also begun to investigate genital divergence within and between species of onthophagine beetles. Pizzo et al. [11] found significant differences in paramere shape among recently diverged natural and exotic populations of *O. taurus*, and these divergences were qualitatively similar to those detected when the same species was compared to its sister species, *O. illyricus*. In contrast, the same study failed to detect corresponding differences in vaginal shape. However, the female vagina does not directly interact with the male parameres during copulation [12], and thus presence/absence of divergence in vagina shape may be insufficient to evaluate the degree to which male and female genitalia may be evolving in concert. Instead, and as emphasized in recent important reviews [5], [13], addressing this issue requires an understanding of the details of morphological fit and interaction between male and female genitalia. Here we quantify and compare morphological evolution of size and shape of male parameres and female pygidial flaps, two structures that do interact tightly during copulation. As detailed in Figure 1, during copulation male parameres fit into pits located internally near the base of the pygidial flap in order to gain stability that subsequently facilitates sperm transfer [12].

We focus on male paramere and female pygidial flap morphology to investigate the patterns of copulatory structure divergence and codivergence between sexes over a range of phylogenetic distances and stages of evolutionary divergence. In particular, we compare and contrast (a) one native and three rapidly diverging exotic populations of *Onthophagus taurus* [11], [14] and its sister species *O. illyricus* [15], and (b) three species belonging to the species complex *fracticornis-opacicollis-similis*, in which *O. similis* and *O. opacicollis* are sister species, whereas *O. fracticornis* is more distantly related [16]. Our study aims (1) to quantify and compare divergence patterns of both male parameres and female pygidial flaps across populations and species, (2) to assess whether size and shape development are correlated within each structure considered, and (3) to evaluate whether size and/or shape of male and female genitalia appear to be diverging in concert.

## Materials and Methods

### Specimens, image acquisition and measurement

Genital association of male and female genitals during copulation have been described for *O. taurus* by Werner and Simmons [12]. After the male positions himself on the back of the female, the latter lifts her pygidium, and the male inserts his parameres under it. At this stage, parameres engage with a specific region of the ventral, inner side of the female pygidium (the folded distal inner border of tergite VIII [17], hereafter referred to as pygidial flap for simplicity: Figure 1). The apices of the parameres are inserted in pits located internally near the base of the pygidial flap, and once a mechanically stable position is gained the male retracts his aedeagus slightly and tilts his body backwards so that male-female physical interactions occur only through the coupling of parameres and pygidial flap. At this point, the endophallus is inflated into the female bursa copulatrix and a spermatophore is passed into the female genital tract.

To explore size and shape divergence patterns of parameres and pygidial flaps in *Onthophagus* beetles, we took into account two model systems: (a) the sister species *O. taurus* and *O. illyricus* (subgenus: *Onthophagus s.s.*) [15] and (b) the species-complex *O. fracticornis-similis-opacicollis* (subgenus: *Palaeonthophagus*) [16], as detailed below.

- We investigated three exotic (Eastern Australia: EA; North Carolina, USA: NC; Western Australia: WA) and one native (Italy: IT) population of *O. taurus*, as well as one Italian population of its sister species *O. illyricus* (ILLY). Analyses of males were conducted on specimens previously used in [10]. Female beetles of the same populations were field-collected and chosen for analyses at random. Collection sites and sample sizes were as follows. EA: Tumut and Cargo, 51♂, 49♀. NC: Orange and Durham Counties, 47♂, 50♀. WA: Narrikup, 48♂, 49♀. IT: Piedmont, 32♂, 49♀. ILLY: Piedmont, 34♂, 33♀. Individuals from Tumut and Cargo (EA) and from Orange and Durham Counties (NC) were considered part of the same panmictic populations on the basis of the lack of geographical barriers between them. 2D images of male pronota and parameres, as well as measurements of pronotum width (used as an estimate of body size: [18], [19]), were acquired by HF Parzer as described in [10]. After being dissected by hand from each specimen, female pygidia were positioned on plasticine supports. Care was taken to ensure that edges were aligned on the same horizontal plane. Pygidial flaps were then photographed using the same morphometric setup as described in [10]. Images of female pronota and pygidial flaps, as well as measurements of pronotum width, were collected by ALM Macagno.
- Specimen of *O. fracticornis* (18♂, 28♀), *O. similis* (19♂, 29♀) and *O. opacicollis* (16♂, 30♀) were collected respectively in Western Italian Alps (Valle d'Aosta), Central France (Auvergne) and Central Italy (Tuscany) [16]. Pronota and copulatory structures were treated as previously described and photographed with a two dimensional image analysis equipment, including a Leica Z16Apo stereoscope and a Leica DFC320 digital camera (Leica Microsystems AG, Wetzler, Germany). All images and measurements (taken with the software LAS v 2.5.0 - Leica Application Suite) were collected by ALM Macagno.

### Geometric morphometrics: landmark acquisition and GPA

**Figure 2:**
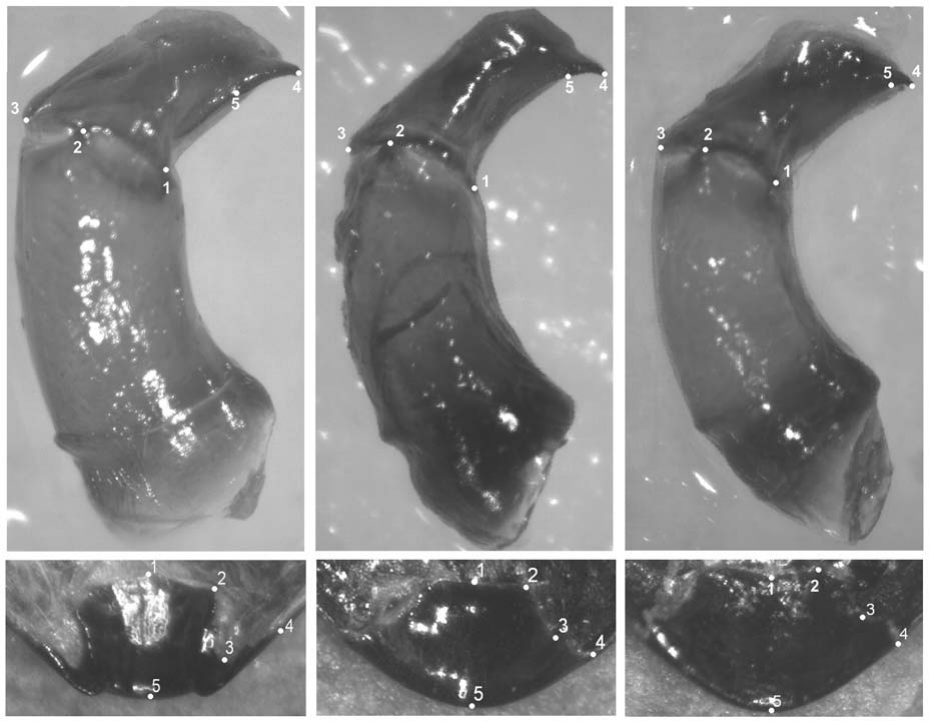
Landmark configurations used to describe the shape of parameres and pygidial flaps in *O. fracticornis* (left), *O. similis* (centre) and *O. opacicollis* (right).

We used a landmark-based geometric morphometric approach [20]–[23] to characterise the form of parameres and pygidial flaps, and to inspect separately their patterns of size and shape variation between populations and species. This analysis was conducted separately for the four populations of *O. taurus* and its sister species *O. illyricus* on one side, and for the species-complex *O. fracticornis-similis-opacicollis* on the other, as we were interested in detecting small differences within and between males and females, which would have been swamped by the huge differences that exist between the two subgenera. With this method, structures are defined by cartesian coordinates of points ( = landmarks) that can be located unambiguously on every specimen, and that correspond in a one-to-one manner from one specimen to another. Landmark configurations used to analyse the two structures are reported in Figure 1 and 2. All landmarks were digitized on the images by the same person (ALM Macagno) using *TpsDig 2.10* [24]. For each structure, the landmark configuration was chosen following criteria of homology in every specimen [20] and detection ease. Specifically, we used Bookstein's type I and type II landmarks, i.e., respectively, points that occur at tissue junctions (and whose homology is therefore based on biological evidence), and points whose homology is supported only by geometric evidence (e.g., points of maximum curvature) [20].

Landmarks were digitized on the left male paramere as well as left half of the female pygidial flap.

We used the Generalized Procrustes Analysis (GPA) to separate geometrical information related to translation, rotation, and scale from information relating to shape only [25]. After Procrustes superimposition, each structure (defined by its landmark configuration) corresponds to a point on a curved, non-Euclidean shape space (the Kendall's shape space: [26], [27]). Data have to be projected onto a Euclidean space tangential to a reference point in Kendall's shape space to allow standard multivariate analyses (which assume linear spaces) of shape variation [28]. To perform this task, we used an orthogonal projection onto the space perpendicular to the vector of shape coordinates of the reference shape [22]. All of the analyses were performed in *MorphoJ* [29]. As long as variation in shape space is small, data in tangent space are an almost perfect approximation of the data in shape space. We tested this approximation using *tpsSmall 1.20* [30].

The centroid size of each structure (i.e., the square root of the sum of squared distances of the set of landmarks defining the structure from their centroid, or centre of gravity [20]) was saved as a separate variable and used as an estimate of size. This measure is approximately uncorrelated with shape for small isotropic landmark variation [20], [22], [31], and was therefore used to estimate size divergence patterns of copulatory structures across species and populations independently from data on their shape.

### Size divergence of copulatory structures

Ln-transformed measurements of centroid size were used as an estimate of the size of parameres and pygidial flaps. To inspect inter- and intraspecific differences of their size relatively to body size, we first computed their static allometries. A preliminary inspection of scatterplots of ln-transformed pronotum width *vs.* ln-transformed centroid sizes of copulatory structures did not reveal any significant deviation from linearity. Therefore, we used method-of-moments Standardised Major Axis (SMA) regressions to fit these distributions [32]. Measurement error variance was computed on ln-transformed data, for each structure and species-group, by re-measuring or replacing landmarks three times on a subset of individuals (25 *O. taurus-illyricus*; 21 *O. fracticornis-similis-opacicollis*) [32]. We first tested for common slope across groups (separately: 4 populations of *O. taurus*, 1 of *O. illyricus*; 1 population for each species of *O. fracticornis-similis-opacicollis*). Given the non-significance of these tests, we used Wald statistic to test for shifts in elevation between groups. Where Wald statistics were significant, we run post-hoc multiple comparisons to assess the significance of elevation differences across groups. All analyses were conducted in *SMATR* [33].

The same allometric data were also fitted with Ordinary Least Squares (OLS) regressions and compared with ANCOVAs. Since OLS and SMA approaches gave similar results, here we only present results obtained with the SMA method [32].

### Shape divergence of copulatory structures

We preliminarily inspected patterns of inter- and intraspecific shape variation of male and female copulatory structures with a PCA of the covariance matrix of shape variables and visualization of deformation grids. We then used Procrustes distances (i.e., the square root of the sum of squared differences between the positions of the landmarks in two optimally superimposed configurations at centroid size [20]) to quantify the degree of shape divergence in pairwise comparisons between groups (separately: 4 populations of *O. taurus*, 1 of *O. illyricus*; 1 population for each species of *O. fracticornis-similis-opacicollis*) [34], and assessed their significance with permutation tests (10,000 permutation rounds). Analyses were conducted in *MorphoJ* [29].

### Congruence of shape and size in the evolution of copulatory structures

We assessed the degree of dependence of copulatory structures' shape on body size for each population and species with multivariate regressions of shape variables of copulatory structures onto ln-transformed measures of pronotum width [35]. The significance of percentages of shape variance explained by body size was assessed with permutation tests against the null hypothesis of independence (10,000 iterations). To inspect the degree of developmental correlation between copulatory structures' shape and size, we used Partial Least Squares analyses (PLS: [23], [31], [36], [37]) aimed at assessing the covariation between shape variables and ln-transformed centroid size of copulatory structures of different species and populations. The strength of association between size and shape of copulatory structures was represented by RV coefficients [38], [39], and their significance was checked with permutation tests (10,000 permutation rounds). All analyses were performed in *MorphoJ* [29].

For both parameres and pygidial flaps, size divergence between populations and species was expressed as the elevation difference of static allometries in pairwise comparisons between groups, whereas shape divergence was represented by Procrustes distances between groups. Divergence measures found in pairwise comparisons were correlated with the Spearman's rank correlation coefficient (*ρ*) to check for signatures of correlated patterns of evolutionary change across populations and species of (i) size and shape of the same structure, (ii) size of male and female copulatory structures and (iii) shape of male and female copulatory structures. Since number and placement of landmarks used to describe parameres and pygidial flaps were different between the two subgenera considered here, correlation analyses with Spearman's *ρ* where only performed on the *O. taurus – O. illyricus* system, which permitted a sufficient number of contrasts. Correlation tests were performed in SPSS.

## Results

### Divergence of copulatory structures within and between species: relative size

Parameters (slope, intercept, and *R* ^2^) of method-of-moments SMA regressions between ln-transformed measures of pronotum width and centroid size of copulatory structures are reported in Table 1. The percentages of variation in copulatory structure size explained by body size were lower in males than in females, except in the case of *O. fracticornis* (*R^2^* in Table 1). Furthermore, the slopes of scaling relationships were in the range of negative allometries (*i.e.*, a<1) for both parameres and pygidial flaps, and were consistantly lower in males than in females (Table 1).

**Table 1:** Static allometries of male and female copulatory structures.

	*O. taurus - O. illyricus*					*O. fracticornis - O. similis - O. opacicollis*		
	Population					Species		
Parameter	EA	IT	NC	WA	ILLY	Of	Os	Oo
♂: Paramere								
*y_0_*	5.70	5.68	5.50	5.43	5.69	2.51	2.43	2.57
*a*	0.44	0.45	0.57	0.61	0.45	0.35	0.48	0.34
*R* ^2^	0.23	0.32	0.47	0.22	0.24	0.53	0.30	0.19
♀: Pyg. flap								
*y_0_*	4.91	4.95	4.92	4.97	4.62	2.02	2.11	2.17
*a*	0.69	0.66	0.69	0.66	0.84	0.90	0.87	0.85
*R* ^2^	0.61	0.48	0.62	0.73	0.72	0.48	0.65	0.77

Parameters of Method-of-moments SMA regressions between ln-transformed measures of pronotum width and centroid size (CS) of each copulatory structure (*a* = slope, *y_0_* = intercept, *R* ^2^). Within sexes, slopes do not differ significantly across populations (*O. taurus - O. illyricus*) or species (*O. fracticornis - O. similis - O. opacicollis*). Pairwise differences in elevation of static allometries are reported in Table 2.

When all four populations of *O. taurus* and the single *O. illyricus* population were compared to each other, both paramere and pygidial flap static allometries exhibited common slopes across groups (test statistic: 5.29, *P* = 0.27; test statistic: 4.45, *P* = 0.36, respectively). Using Wald statistics, we detected no significant differences across groups in the elevation of paramere static allometries (test statistic = 4.46, *P* = 0.35), but a significant elevation shift of pygidial flap allometries (test statistic = 80.86, *P*<0.001). Subsequent pair-wise comparisons of intercepts (sequential Bonferroni corrections applied) highlighted widespread significant intra- and interspecific size divergences in females (Table 2). Most notably, relative pygidial flap size was significantly smaller in *O. illyricus* compared to all of *O. taurus* populations considered, though interestingly, paramere size did not differ between the two species or within *O. taurus* populations. Similarly, in the *O. fracticornis-similis-opacicollis* species complex, static allometries of paramere and pygidial flaps exhibited common slopes across groups (test statistic: 1.73, *P* = 0.44; test statistic: 0.11, *P* = 0.95, respectively), but significant size divergences (i.e., elevation shift of allometries) were detected (paramere: Wald statistic = 264.39, *P*<0.001; pygidial flap: Wald statistic = 320.12, *P*<0.001). Specifically, *O. opacicollis* had substantially larger parameres than *O. similis* and *O. fracticornis* (the latter two being of comparable size). *O. opacicollis* also exhibited the largest pygidial flaps, followed by *O. similis* and *O. fracticornis*.

**Table 2:** Size and shape divergence of copulatory structures between populations and/or species.

Comparison	♂: Paramere		♀: Pygidial flap	
	Shape div.	Size div.	Shape div.	Size div.
ILLY-EA	0.068**	ns	0.092**	0.06**
ILLY-NC	0.077**	ns	0.086**	0.05**
ILLY-IT	0.071**	ns	0.078**	0.03**
ILLY-WA	0.067**	ns	0.073**	0.07**
WA-EA	0.036**	ns	0.027**	ns
IT-EA	0.032**	ns	ns	0.03*
IT-NC	ns	ns	0.020*	(0.02*)
WA-NC	0.022*	ns	ns	ns
EA-NC	0.036**	ns	0.021*	ns
IT-WA	0.025*	ns	ns	0.03**
Of-Os	0.157**	ns	0.241**	0.07**
Of-Oo	0.187**	0.06**	0.368**	0.11**
Os-Oo	0.055**	0.06**	0.130**	0.04**

Shape divergence is represented by Procrustes distances between groups (significance was assessed with 10,000 permutations rounds). Size divergence is expressed as the elevation difference between static allometries of parameres and pygidial flaps in pairwise comparisons between groups; only divergences that were significant (sequential Bonferroni correction applied) are reported. The comparison between pygidial flap size of IT and NC was significant after removing *O. illyricus*.

**P<0.01;

*P<0.05; ns = not significant.

### Divergence of copulatory structures within and between species: shape

**Figure 3:**
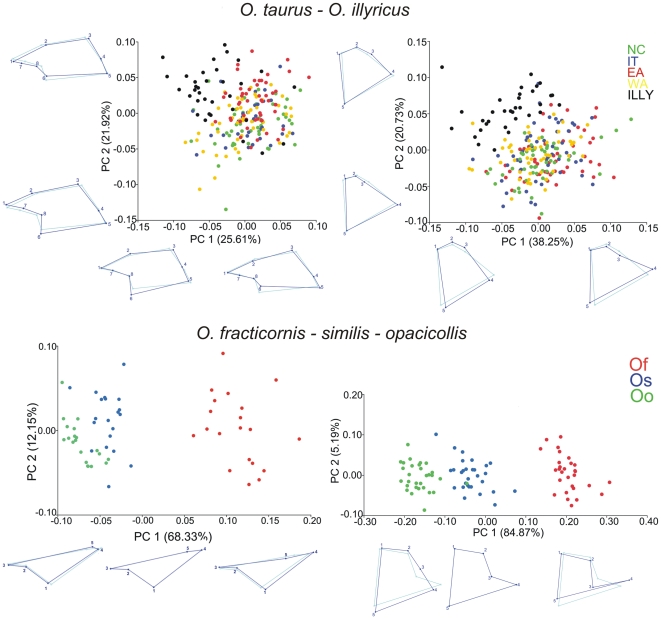
Scatterplot of shape of male parameres (left) and female pygidial flaps (right) according to principal component analyses of covariance matrices. Wireframe graphs show shape modifications (dark blue lines) with respect to the consensus shape (light blue lines) of the copulatory structures as described by the correspondent PC axes.

Results of the PCA conducted on parameres and pygidial flaps and deformation grids pertaining to PC1 and PC2 are shown in Figure 3, and the significance of Procrustes distances across groups in analysis are reported in Table 2. *O. illyricus* exhibited considerable divergence in paramere and pygidial flap shape compared to the four *O. taurus* populations. Inspection of deformation grids showed that male *O. illyricus* had parameres that appeared stockier and stouter compared to those of *O. taurus*, which in turn appeared more elongated. At the same time, the pygidial flap of female *O. illyricus*, and in particular the distal portion, where the apex of paramere is inserted during copulation (as defined by landmarks 1-2-3), appeared considerably wider in *O. illyricus* than in *O. taurus*. Small, although significant, shape differences between populations of *O. taurus* (e.g. in the comparison between EA and WA) also showed a widening of the distal part of the pygidial flap in populations where parameres had a squatter shape. The species of the complex *O. fracticornis-similis-opacicollis* likewise exhibited substantial shape divergence of both male and female copulatory structures. *O. fracticornis* had a longer apex of the paramere, and a pygidial flap with a conspicuous central prominence. In *O. opacicollis* and *O. similis*, which in contrast were more similar in shape, parameres had shorter apices, and pygidial flaps showed a more gradual connection with the ventral border of the pygidium.

### Independent evolution of size and shape of copulatory structures

Multivariate regression of shape variables of copulatory structures onto ln-transformed measures of pronotum width showed that both paramere and pygidial flap shape variations were substantially independent from body size in all the species and populations analysed. Permutation tests against the null hypothesis of independence (10,000 iterations) yielded no significant results (*P*>0.05) for *O. fracticornis*, *O. similis*, *O. opacicollis* and *O.illyricus*, and for *O. taurus* populations collected from EA, IT, and NC. The only exception were *O. taurus* collected in WA, for which the null hypothesis of independence was rejected in both females (*P*<0.01) and males (*P* = 0.02). Here, however, the percentage of shape variation of copulatory structures explained by body size was very low (7.53% and 5.75%, respectively).

RV coefficients that express the covariation between shape and size (estimated by ln-transformed centroid sizes) of copulatory structures and derived from PLS analyses of males and females in different populations and species are reported in Table 3. RV coefficients can range from 0, if the two sets of variables in analysis are completely independent, to 1, if two sets are completely interdependent. In our analyses, RV coefficients highlighted as significantly different from 0 remained very low overall.

**Table 3:** Developmental correlation between size and shape of copulatory structures.

Population	RV coefficient	
	♂: Paramere	♀: Pyg. flap
EA	0.14**	0.02 ns
IT	0.04 ns	0.04 ns
NC	0.14*	0.02 ns
WA	0.19**	0.13*
ILLY	0.12 ns	0.14*
Of	0.11 ns	0.13 ns
Oo	0.08 ns	0.08 ns
Os	0.08 ns	0.03 ns

RV coefficients (range: 0–1) express covariation between centroid size (ln transformed measurements) and shape of copulatory structures as determined with PLS analyses of the eight groups. Significance of RV coefficients was assessed with permutation tests (10,000 permutation rounds).

**P<0.01;

*P<0.05; ns = not significant.

In *O. taurus* and *O. illyricus*, size (expressed as the elevation difference between static allometries in pairwise comparisons) and shape divergence (expressed as Procrustes distances) of male parameres across populations (see Table 2) appeared completely uncorrelated, to the point that parameres of *O. illyricus* diverged from the four populations of *O. taurus* in shape but not in size. Similarly, we did not detect any significant parallels between size and shape divergence of female pygidial flaps (Table 2) (Spearman's *ρ* = 0.55, *P* = 0.10), with the exception of *O. illyricus* which diverged from the four populations of *O. taurus* in both pygidial flap size and shape. As for *O. fracticornis-similis-opacicollis*, size and shape of parameres showed an incongruent pattern of variation, whereas both size and shape of pygidial flaps diverged more in *O. fracticornis*, with *O. opacicollis* and *O. similis* remaining more similar (Table 2). Combined, our data thus suggest that size and shape of male and female copulatory structures can evolve rather independently from one another across different populations and species.

### Co-variation of shape but not size of male and female copulatory structures

Table 2 summarizes all constrasts executed to compare divergence patterns of size and shape between male and female copulatory structures. The same condition of size divergence (presence vs absence) between males and females *O. taurus* and *O. illyricus* was detected in only three out of ten contrasts (Table 2). *O. illyricus* did not diverge in paramere size from any of the *O. taurus* populations, and therefore, overall, the size divergence patterns of parameres and pygidial flaps were largely incongruent. In partial contrast, both male and female copulatory structures of *O. opacicollis* diverged significantly in size from both *O. fracticornis* and *O. similis.* The latter two species also differed significantly from each other in pygidial flap size, but not in male paramere size.

As for the shape of copulatory structures, both male *and* female *O. illyricus* diverged significantly from *O. taurus*, and six out of ten contrasts (Table 2) detected a corresponding pattern of shape divergence between male and female copulatory structures across populations and species (Spearman's *ρ* = 0.87, *P*<0.01). *O. fracticornis-similis-opacicollis* showed a fully congruent pattern of shape variation for male parameres and female pygidial flaps, with *O. fracticornis* being more differentiated from the other two species, which were in turn more similar. Combined, our results suggest that male and female copulatory structures appear to be diverging in concert with respect to shape, but to a much lesser degree, if at all, with respect to size.

## Discussion

Genitalia are among the fastest evolving morphological traits in arthropods [1]. Thorough testing of many of the hypotheses aimed at explaining this observation requires detailed knowledge of the interactions between male and female copulatory structures during mating, data rarely available for the vast majority of species [5]. Here we compared and contrasted size and shape evolution of male parameres and female pygidial flaps (i.e., two structures known to interact tightly during copulation) across populations and species of onthophagine beetles. Comparisons within each sex showed that shapes and sizes of both structures diversify readily - though largely independently of each other - within and between species. Similarly, comparing patterns of covariation across sexes showed that relative sizes of male and female copulatory organs evolve largely independent of each other. However, performing this analysis for genital shape revealed a signature of parallel divergence. Our results therefore suggest that male and female copulatory structures that are linked mechanically during copulation may diverge in concert with respect to their shapes, but to a much lesser degree, if at all, with respect to size. Furthermore, our results suggest that genital divergence in general, and codivergence of male and female genital shape in particular, can evolve between closely related species and even recently established populations, and thus in a remarkably short amount of time. Below we discuss the most important implications of our results.

### Male and female copulatory structures diverge rapidly between populations and species

At every level of phylogenetic relationship examined in this study (species complex, sister species, recently established populations) we found evidence for widespread significant divergences in the shapes and, to a lesser degree, sizes of male parameres and female pygidial flaps. These results both confirm and critically extend previous studies. Specifically, they confirm previous results from a pilot study on genital evolution in *O. taurus* which provided the first evidence that paramere morphology has diverged across recently established exotic populations [11]. Our results extend this and other previous studies by demonstrating that this observation holds true beyond these populations and also applies to female copulatory structures that tightly interact with parameres during copulation. Specifically, we found that - like male parameres - female pygidial flaps exhibited extensive divergence in shape, and again to a lesser degree in size, across species and populations. Combined, these results provide support for hypotheses on genitalia evolution that implicitly or explicitly predict concomitant evolutionary changes of male and female genitalia (see below).

### Genital shape evolves faster, and independent of, genital size

Most of the divergences of copulatory structures we detected in this study occured in shape and much less so in size, with only two exceptions: female *O. taurus* collected in Italy diverged from those collected in Eastern and Western Australia in size of pygidial flap, but not in shape. Apart from these cases, however, genitalic shape evolved generally faster, and independent of, genitalic size. Rapid interspecific genitalic divergence has generally been attributed to shape, rather than size, variation [40], and a number of studies have emphasized the evolutionary independence of genitalic shape and size [41]–[43]. Most importantly, Simmons et al. [8] demonstrated in *O. taurus* that aedeagus shape can diverge extremely rapidly in response to directional sexual selection in the lab, whereas size remained unaffected in the process. In addition, recent studies provided evidence for mosaic evolution of genitalia [12], [44], [45], suggesting that size and shape of different portions of the same copulatory structures may respond to different selective pressures depending on their function during copula. Together, these findings suggest that size and shape of genital structures and their component parts are developmentally and genetically decoupled enough to evolve independent of each other. Unfortunately, the developmental and genetic mechanisms underlying the regulation of genital form are relatively poorly understood in insects [10], [43], [46] though what is known suggests that many of the same developmental genetic processes that take place in appendages such as legs and mouthparts also contribute to genital differentiation [47]–[49]. If correct, this suggests that local, segment specific regulation of differential growth and differentiation must underlay genitalic development and evolution, rather that genitalia-specific developmental processes. Neither scenario, however, can explain the relative paucity of genital size evolution detected here and in other studies. Genital size may be less evolutionarily labile because it is under stronger stabilizing selection, e.g. due to selective mechanisms like ‘one size fits all’ [4] which, along with mechanical fit [5], are thought to underlay the low allometric slopes found in both male and female copulatory structures. Alternatively, genital size may evolve slowly because of developmental constraints imposed by the growth of other structures. For instance, a growing number of studies suggest that insect appendages, including male genitalia, trade-off during development, and that such tradeoffs influence allocation decisions during immature stages [9], [10]. If correct, this raises the possibility that genitalia size in particular, and appendage size in general, may be under greater pleiotropic constraints than shape, and thus less likely to diverge quickly between populations and closely related species.

### Parallels in shape (but not size) divergence between male and female genitalia

We detected a significant degree of codivergence between the shapes, but not sizes, of male and female copulatory structures (Table 2). Specifically, we found a concerted pattern of male *and* female genital divergence in six out of ten contrasts (Table 2) across the four populations of *O. taurus* and one of *O. illyricus*, compared to three out of ten for size. Similarly, while the shape divergence patterns in the *O. fracticornis-similis-opacicollis* complex were fully congruent, the ones of size were not (*O. fracticornis* diverged from *O. similis* in size of pygidial flaps, but not in size of parameres). On one side, this is in line with the higher level of evolvability documented separately for male and female genitalia shape compared to size as discussed above. On the other, it is consistent with concerted divergence of male and female genital shape.

Importantly, such correlations in the degree of divergence among species or populations may simply reflect overall cumulative divergences in morphology, rather than co-evolved differences specific to interacting genitalic traits. However, this is unlikely to be the case here, for two reasons: first, native and introduced *O. taurus* and *O. illyricus* populations show no obvious shape divergence of external traits [11]. Second, a previous study on the same populations yet focused on the vagina – a tract of the female genital apparatus that does *not* engage in physical contact with male genitalia during copulation – also failed to find any evidence for shape divergence among native and introduced *O. taurus* populations [11].

Interestingly, within the *O. fracticornis-similis-opacicollis* species complex, shapes of both male and female copulatory structures did diverge in keeping with the phylogeny of the complex [16], suggesting that here simple phylogenetic dependence may have driven the divergence patterns highlighted in our study. However, it is noteworthy that *sizes* of male and female copulatory structurs did *not* diverge in keeping with phylogeny [16], supporting the general pattern that male and female genital shape is more likely to diverge in parallel than is size. Combined, our data thus suggest that male and female genital shape may codiverge measurably even over time frames as short as those separating *O. taurus* populations (<100 generations: [9]) or across closely related taxa within species-complexes such as the *O. fracticornis-similis-opacicollis* complex [16]. More generally, our results provide support for hypotheses that assume or predict concomitant changes of male and female genitalia, specifically lock-and-key, sexual conflict, sexual selection by cryptic female choice, and pleiotropy.

For instance, according to the lock-and-key hypothesis, male and female genitalia are expected to coevolve to ensure effective sperm transfer and minimize heterospecific matings. McPeek et al. [50] found evidence of such a pattern of concerted evolution between male cerci and female thoracic plates in a damselfly genus, concluding that the interaction of those structures is crucial to pre-mating reproductive isolation. *Onthophagus* parameres are coupled with pygidial flaps at the beginning of copulation, in a way that assures mechanical stability and allows correct inflation of the endophallus into the female genital tract [12]. Although it is not known whether the pygidial flap could act as a proper ‘lock’ able to exclude heterospecific matings, it appears likely that proper coupling of the two structures can favor efficient sperm transfer, making heterospecific matings less effective. Interestingly, as in the case of *Enallagma* damselflies [51], the degree of differentiation we found between populations and species was higher for shape than for size of the structures analysed, suggesting that shape might be the main morphological component used for species recognition.

Concomitant evolutionary changes of male and female structures are also expected in the sexual conflict hypothesis, according to which male adaptations that increase control over reproduction by causing damage to females are counteracted by female adaptations to reduce such damage [52], [53]. Sexual selection has been convincingly implicated as a major force underlying the fast divergence of parameres in *O. taurus* [8]. The occurrence of female *Onthophagus* with broken pygidial flaps in collection materials (Macagno, pers. obs.) raise the possibility that correlated shape divergences across sexes could actually be the result of sexual conflict.

Cryptic female choice of paramere shape could also result in a concerted pattern of evolution via runaway selection. Under this hypothesis, the morphology of female copulatory traits affects how females perceive stimuli during copula, and females select sperm of males that provide the right tactile stimuli during copulation due to their genital morphology [13]. It is therefore expected that any changes in female copulatory traits would affect the function of male copulatory structures accordingly. However, this scenario also implies that (1) anatomical areas that are coupled during copulations carry specific receptors that allow females to detect tactile stimuli and (2) these stimuli are perceived differently depending on the shape of male and female copulatory structures. Presently, no data are available that support these inferences.

Lastly, concomitant changes of male and female genital morphology is also expected under the pleiotropy hypothesis, which posits that male and female genitalia share some of their genetic basis with other structures. Evolutionary changes in these structures may therefore bring about correlated evolutionary changes in male and female genitalia. Critical evaluation of this hypothesis is handicapped by a generally poor understanding of the developmental genetic basis of genitalia. However, a growing number of studies show that genetic manipulations directed at appendage development generally also affect genitalic growth (e.g. insulin signaling: Snell-Rood and Moczek, in review) and differentiation (proximo-distal patterning: [54]; TGFβ signaling: [55]). This suggests that, in principle, much developmental opportunity exists for pleiotropy-driven genitalic divergence and coevolution. Future studies on the interactions between male and female genitalia during copulation, and on the development of size and shape of different parts of copulatory structures of both sexes, will be essential to further our understanding of the evolution of genital diversity.
